# Effects of 8 weeks parent-accompanied swimming on physical capacity and intelligence in preschool children

**DOI:** 10.3389/fpubh.2024.1410707

**Published:** 2024-05-31

**Authors:** Yichao Yu, Lei Xia, Huiping Yan, Yifan Lu

**Affiliations:** ^1^The School of Sports Coaching, Beijing Sports University, Beijing, China; ^2^Laboratory of Sports Stress and Adaptation of General Administration of Sport, Beijing Sport University, Beijing, China; ^3^Shenzhen School Affiliated to Sun Yat-sen University, Shenzhen, China; ^4^The School of Sports Medicine and Rehabilitation, Beijing Sports University, Beijing, China

**Keywords:** swimming, children health, physical capacity, intelligence, physical exercise

## Abstract

This study aimed to explore the potential effects of 8-week parents-accompanied swimming on the physical capacity and intelligence of preschool children in China. Thirty-six boys (mean age 3.56 ± 0.27 years) were divided into three groups: the traditional physical exercise group (TP, *n* = 12), the accompanied swimming group (AS, *n* = 12) and the independent swimming group (IS, *n* = 12). Participants’ physical capacity was assessed before and after the intervention using the following indicators: height, weight, distance of tennis ball throw, standing long jump distance, time for the 10-meter shuttle run, time for a two-legged continuous jump, sit-and-reach distance, and time on the walking balance beam. Intelligence was assessed at three points: pre-test, mid-test after 4 weeks, and post-test. Data were analyzed using a two-way repeated measures ANOVA, Bonferroni test (*p* < 0.05) and effect size. The time of the AS and IS groups to walk the balance beam was significantly lower than the TP group, with a difference of 1.81 s (*p* < 0.01, [95% CI −3.22 to −0.40], ES = 1.53) and 1.25 s (*p* < 0.05, [95% CI −2.66 to 0.16], ES = 0.81). At the mid-test, the IQ scores of the TP group were lower than the AS group (*p* < 0.05, [95% CI −12.45 to −0.96], ES = 0.89). Additionally, at post-test, the IQ scores of the TP group were significantly lower than those of both AS (*p* < 0.01, [95% CI −14.12 to −2.74], ES = 1.15) and IS groups (*p* < 0.01, [95% CI −12.53 to −3.31], ES = 1.21). Swimming enhances children’s balance and IQ scores more than traditional physical exercises. Involving parents in swimming leads to a more significant increase in IQ scores within 4 weeks of initial swimming exercise.

## 1 Introduction

Childhood development largely influences a person’s future development and determines the rate of growth and development during adolescence. It is vital in establishing human ethics and defining the concept of social progress (1). Regular physical exercise has been demonstrated to improve physical fitness and lower the disease risk within the population (2). Swimming is a popular physical exercise that improves the body’s aerobic capacity and is widely recognized for maintaining good health (3). Previous research has proven the benefits of swimming on children’s health. It demonstrates that swimming can stimulate the existing reflexes in children and generate new neurological reflexes. Furthermore, it improves the speed of nervous system development. Swimming is an effective way to stimulate the development of bones, muscles, and other tissues. It enhances multi-sensory participation and improves the formation of future swimming skills, promoting the whole body and mind development of children, as well as enhancing immunity (4–8). Nevertheless, sub-standard water quality in children’s swimming can lead to rashes, asthma, and other health problems in them (9, 10).

Physical capacity is determined by both hereditary and acquired factors. Individuals show variations in physical traits under heredity, but these traits can also be fortified and developed through acquired physical activities. Experts commonly measure physical capacity using metrics such as cardiorespiratory endurance, muscular strength, and body composition (11). Previous studies have demonstrated that swimming effectively enhances the physical capacity of children. Duncan and colleagues (12) discovered that long-term swimming training caused modifications in children’s body morphology and function, particularly in their height growth. A study of Chinese children has demonstrated that swimming effectively improves boys’ aerobic and cardiorespiratory capacities (13). This finding has been corroborated by the results of other studies as well (14). Besides, further research has shown that swimming positively affects both the cardiorespiratory health and body composition of healthy children (15).

The intelligence quotient (IQ) is a commonly used cognitive ability evaluation. IQ is defined as the relatively stable psychological characteristics of an individual’s understanding of objective things, including a combination of perceptual observation, memory, imagination and thinking (1). Intelligence in the preschool years is a continuous developmental process, it can influence academic achievement, knowledge acquisition, and labor skill levels in young adulthood (16). Physical exercise improves children’s cognitive ability and IQ scores (17, 18). Borioni et al. (19) found significant benefits for children’s language skills after a swimming exercise; Nissim et al. (20) found significant improvements in arithmetic and logical reasoning in preschoolers after 6 months swimming exercise; other studies have also shown that learning to swim improves preschoolers’ intelligence scores and can help reduce the number of delinquent behaviors in children (21).

Developing good physical exercise habits is key to promoting young children’s growth and development. Nowadays, more and more children start learning to swim before learning to walk. Current swimming exercise methods for children can be categorized as the independent swimming and accompanied swimming. The main difference between these two types of workouts is whether or not the parents are involved in the swim workout. As mentioned earlier, there have been many studies exploring the effectiveness of swimming exercise. But the research on these two swimming exercise methods on children’s health was very limited, more empirical research data is needed to fill research gaps in this area.

Therefore, this study aimed to examine the impact of 8 weeks parent-accompanied swimming on the physical capacity and IQ scores of preschool children. We hypothesized that 8 weeks of parent-accompanied swimming more effectively improve physical capacity and intelligence in preschool children.

## 2 Materials and methods

### 2.1 Subjects

This study recruited children between August 2021 and October 2021 from three kindergartens in Xiqing District, Tianjin, China. For this study, participants comprised boys between 3 and 4 years old. All participants were in good health with no recent physical complaints. Based on the calculation of the required sample size according to G*Power (3.1.9.4; Germany), the F test and repeated measure ANOVA, main effects, and interaction effects were selected the prespecified effect size was chosen as 0.30, and the statistical test power 1-β was 0.8 and α was taken as 0.05, 30 healthy boys needed to be recruited to participate in the study.

To prevent subject attrition during the study, 36 healthy children were recruited. All children in the study completed the test without sample loss (Table 1). At kindergarten parent meetings, the test items, experimental protocols, and associated risks were explained to the parents by the researchers. Parents of all children signed informed consents form before participating in the study, clearly comprehended the risks associated with the intervention and volunteered to participate. The study adhered to the Declaration of Helsinki, and the Ethics Committee of Exercise Science at Beijing Sport University approved the study protocol (Grant no. 202008211).

**Table 1:** Basic information of subjects (*n* = 36).

Indicators	
Age (yrs)	3.56 ± 0.27
Height (cm)	106.73 ± 4.39
Weight (kg)	18.33 ± 1.45
BMI (Kg / m^2^)	16.16 ± 1.72

### 2.2 Study design

Participants were randomized assigned into groups based on different physical exercise methods, including one control group: the traditional physical exercises group (TP, *n* = 12) and two experimental groups: the accompanied swimming group (AS, *n* = 12), the independent swimming group (IS, *n* = 12). The AS group’s children only participated in swimming exercises with their parents, while the IS group’s children only participated independently, these two groups received the same swimming exercise interventions. The TP group only participated in traditional physical exercises practiced in kindergarten. Each group of children was exposed to 50 min of physical training twice weekly for 8 weeks. The content of the swimming exercises was developed based on opinions from professional swimming teachers who specialized in training children. The exercises included floating, breathing, leg kicks, paddling and other essential swimming techniques. Table 2 illustrates the detailed teaching content and schedule followed for the swimming exercise. The training content for TP group was developed by physical education teachers and experts, which ensured the physical exercise volume of the children in all three groups were comparable (22). In order to ensure the accuracy and validity of the test, quality control measures were implemented for personnel involved in testing, teaching, data collection, and other related processes. All participants and teachers involved in the test were stabilized and trained, and the parents of the AS group underwent brief training before the start of the intervention on verbal guidance and precautions in effective teaching implementation.

**Table 2:** The specific teaching content and schedule of different groups.

Group	Phase	Content	Time
AS IS	Warm-up part	1.Children get into the water 2.Self-introduction 3.Warm-up	5-8 min
	Basic part	1.Breathing movements and exercises 2.Leg movements and exercises 3.Arm movements and exercises 4.Practicing back float movement	35-40 min
	Relaxation part	1.Course Summary 2.Relaxation 3.Out of water	3-5 min
TP	Warm-up part	1.Self-introduction 2.Warm-up	5-8 min
	Basic part	1.Upper body movements and exercises 2.Trunk movements and exercises 3.Lower body movements and exercises 4.Group game	35-40 min
	Relaxation part	1.Course summary 2.Simple stretching and relaxation	3-5 min

AS, accompanied swimming group; IS, independent swimming group; TP, traditional physical exercise group.

**Figure 1:**
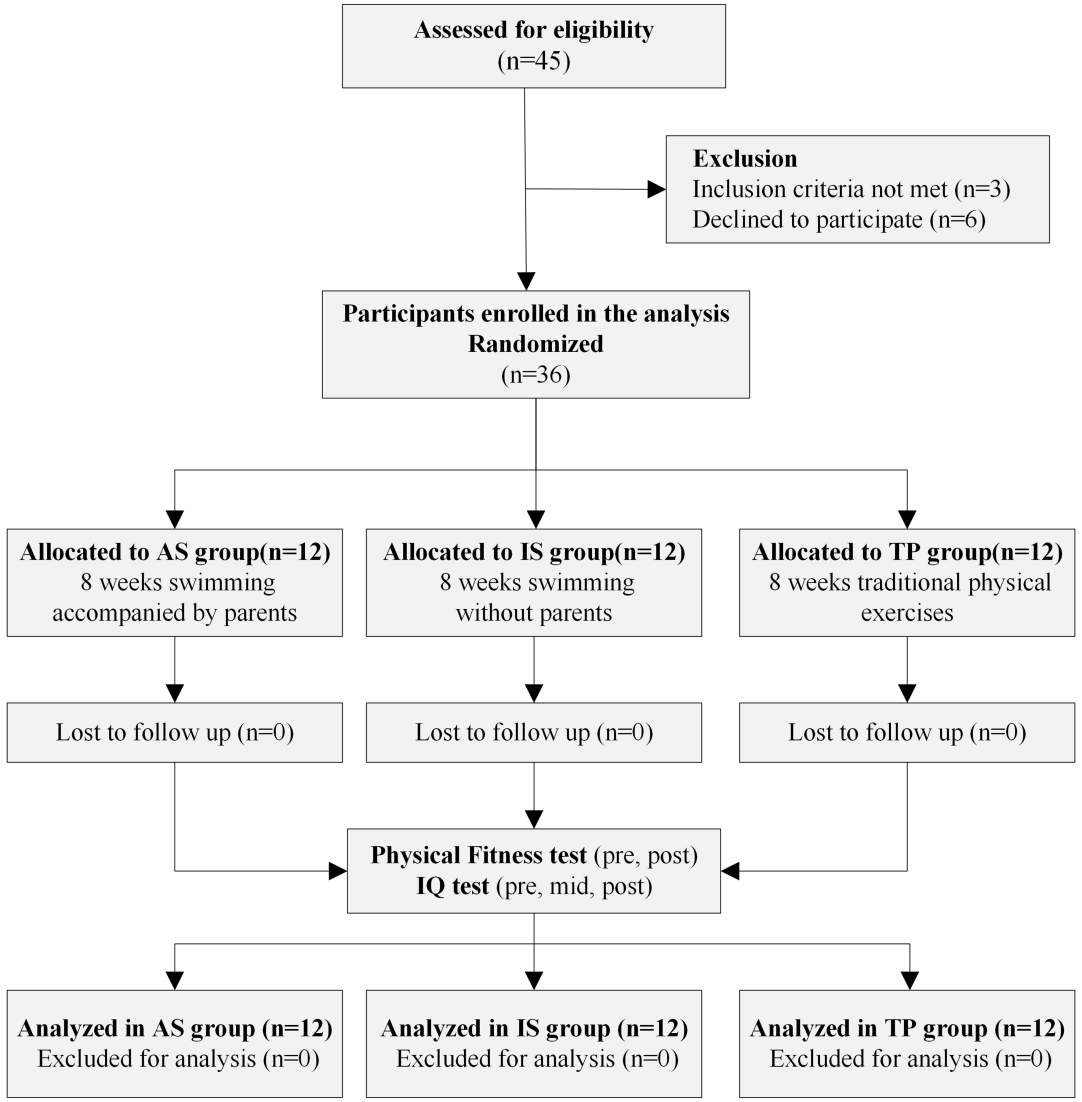
Flow chart of this study. TP, traditional physical exercise group; AS, accompanied swimming group; IS, independent swimming group.

The flow chart of this study as depicted in Figure 1. Physical capacity was tested before and after exercising. Physical capacity tests included height, weight, tennis ball throwing distance, standing long jump distance, 10-meter toss time, two-legged continuous jump time, seated forward bend distance, and walking balance beam time. The IQ were measured in three timepoints (pre, mid and post of intervention). The IQ scores were obtained using the Chinese version of Wechsler Young Children Scale of Intelligence (C-WYCSI) (23).

### 2.3 Test program

#### 2.3.1 Physical capacity test

Physical capacity testing of the participants was conducted using the Chinese National Physical Fitness Standards for Childhood Component (24). The physical capacity test indicators include height, weight, tennis ball throwing distance, standing long jump distance, 10-meter shuttle run time, two-legged continuous jump time, sit and reach distance, and walking balance beam time. The test content is shown in Table 3.

**Table 3:** Content of physical capacity test.

Test	Content
Height and weight	The National Physical Fitness Monitoring Comprehensive Physical Assessment System (GMCS-IV Physical Fitness Test Instrument, Jianmin, Guangzhou, China) was used to measure height and weight. During the test, children were required to stand barefoot on the tester and their height and weight were measured in turn. Height was recorded in centimeters with two decimal places. Weight was recorded in kilograms with two decimal places.
10-meter shuttle run	A stopwatch (S141-Stopwatch, SEIKO, Tokyo, Japan) recorded the results of the 10-meter return run. The test was performed by a group of two people, children set off when they hear the signal from the tester. Starting from a standing position, with the children turning quickly to the target line by touching the return marker with their hand and any part of their body running across the finish line, the timing will be finished. The test was performed twice and the fastest performance was used for analysis. The record was made in seconds, and one decimal point was retained.
Standing long jump	Subjects stood with their feet naturally open, stood behind the starting line, not exceeding the starting line, pre-swing their hands back and forth, jumped to the furthest point with both hands and feet coordinated and forceful, and measured the straight distance between the two points. Tested twice using a standard meter tape and the best performance was used for analysis, recorded in centimeters and retain two decimal places.
Tennis ball throwing	The participant threw the ball with the feet open in front of and behind the head, holding it in one hand above the head and throwing it as far forward as possible. The valid score was the straight distance between the line of the throw and the point where the ball first lands. Tested twice using a standard meter tape and the best performance was used for analysis, recorded in meters and retain two decimal places.
Two-legged continuous jump	Placed 10 soft square packs in a straight line on a flat surface at 50 cm intervals, starting 20 cm from the first soft square pack and ending 20 cm from the 10th soft square pack. The subject stood behind the starting line with both feet together and jumped with both feet at the same time after hearing the command “start,” and the timing ended when the child passed the finish line. The record was made in seconds, tested twice using a standard meter tape and the best performance was used for analysis. One decimal point was retained.
Sit and reach	Using the National Physical Fitness Monitoring Comprehensive Physical Assessment System (GMCS-IV Physical Fitness Test Instrument, Jianmin, Guangzhou, China) to measure sit and reach distance. The test was performed with the subject’s hands together, palms down and flat, knees straight, torso bent forward, and using the tips of the middle fingers of both hands to push the vernier smoothly forward. Tested twice and the best performance was used for analysis, recorded in centimeters and retain two decimal places.
Walking balance beam	The subject stood on the balance beam platform with arms held sideways. The timing started when the “start” was heard and ended when any of the subject’s toes exceeded the endpoint. The record was made in seconds, tested twice and the fastest performance was used for analysis. One decimal point was retained.

#### 2.3.2 Intelligence quotient test

Subjects’ IQ scores were obtained using the C-WYCSI (23). The test was completed by two trained testers, one to coordinate the process and one to administer the test, during which the tester was required to accurately communicate and encourage the children to complete the test, but not to suggest or remind. Before the test, the subjects stayed in a relatively quiet environment, and the whole testing process was not interfered with by other factors. The testers asked questions individually, observed and recorded the subjects’ expressions and answers, and the IQ test was completed after all the questions had been answered. The specific content of the test is shown in Table 4.

**Table 4:** Content of intelligence test.

Test classification	Content
Knowledge test	knowledge about the child’s body, the concept of time, general knowledge of daily life, nature, personnel relations, and measurement relations.
Picture vocabulary test	The test material consists of four pictures in which the tester says a word and asks the child to point out one of the four pictures that fits the meaning of the word.
Picture filling test	Children are asked to recognize the contents of the picture and to identify the missing parts of the picture.
Wooden block pattern test	Children were asked to pose the same pattern as the samples in different samples for a specified period of time.

The IQ test comprises four sections: a knowledge test (with a maximum score of 23), a picture vocabulary test (with a maximum score of 44), a picture filling test (with a maximum score of 25), and a wooden block pattern test (with a maximum score of 29). The marks for the knowledge test, picture vocabulary test, and picture filling test were rated on a scale of 1 for each correct answer and 0 for an incorrect response. The wooden block pattern test scores were based on time, marked on a scale of 0 to 4 according to how quickly the children solved it. Raw scores were converted to standard scores, following the instruction manual, after examination. IQ was calculated by weighing the scores of each subtest, following the instruction manual (normal mean = 100, SD = 15.0) (25).

### 2.4 Statistical analyses

All data were summarized and collated using Excel 2019 software, and statistical analyses were performed using SPSS 26.0 software (IBM Corp. Armonk, NY, United States). The Shapiro–Wilk test was used to analyze the normal distribution of the data, and all data were presented as mean ± standard deviation (SD). A two-way repeated measures ANOVA was used to determine the effect of time and group on physical capacity indicators (height, weight, 10-meter dash, standing jump, tennis ball throw, two-legged continuous jump, seated forward bending and walking on the balance beam) and IQ test scores and a Bonferroni *post-hoc* corrections test was applied for two-by-two comparisons. The significance level was set at *p* < 0.05, and the high significance level was set at *p* < 0.01. ES values were evaluated according to the following criteria: 0 to 0.19 trivial, 0.20 to 0.59 small, 0.6 to 1.19 moderate, 1.20 to 1.99 large, ≥2.00 very large.

## 3 Results

### 3.1 Results of the effect of swimming on the physical capacity

All data in each group obeyed a normal distribution by the Shapiro–Wilk test (*p* > 0.05). A one-way ANOVA test found homogeneity among the three groups in Pre-test (*p* > 0.05). The results of the spherical test showed that *p* < 0.02, which did not meet the spherical test. The results were calibrated in one-way ANOVA “Greenhouse–Geisser.” The results of the calculation of the effect relationship are shown in Table 5.

**Table 5:** The results of two-factor repeated measures ANOVA.

Indicators		Time *group	Time	Group
Height (cm)	F	0.577	158.336	0.213
	p	0.567	< 0.001	0.809
	η^2^	0.034	0.828	0.013
Weight (kg)	F	3.901	271.128	0.053
	p	0.030	< 0.001	0.948
	η^2^	0.191	0.891	0.003
10-meter shuttle run time (s)	F	1.010	192.243	3.248
	p	0.375	< 0.001	0.052
	η^2^	0.058	0.853	0.164
Standing long jump (cm)	F	10.401	273.057	0.107
	p	< 0.001	< 0.001	0.899
	η^2^	0.387	0.892	0.006
Tennis ball throwing distance (m)	F	13.419	362.580	0.158
	p	< 0.001	< 0.001	0.855
	η^2^	0.449	0.917	0.009
Two-legged continuous jump time (s)	F	19.461	264.083	0.254
	p	< 0.001	< 0.001	0.777
	η^2^	0.541	0.889	0.015
Sit and reach (cm)	F	37.021	684.748	0.240
	p	< 0.001	< 0.001	0.788
	η^2^	0.692	0.954	0.014
Walking balance beam (s)	F	2.670	132.902	1.842
	p	0.005	< 0.001	0.174
	η^2^	0.273	0.801	0.100

**Figure 2:**
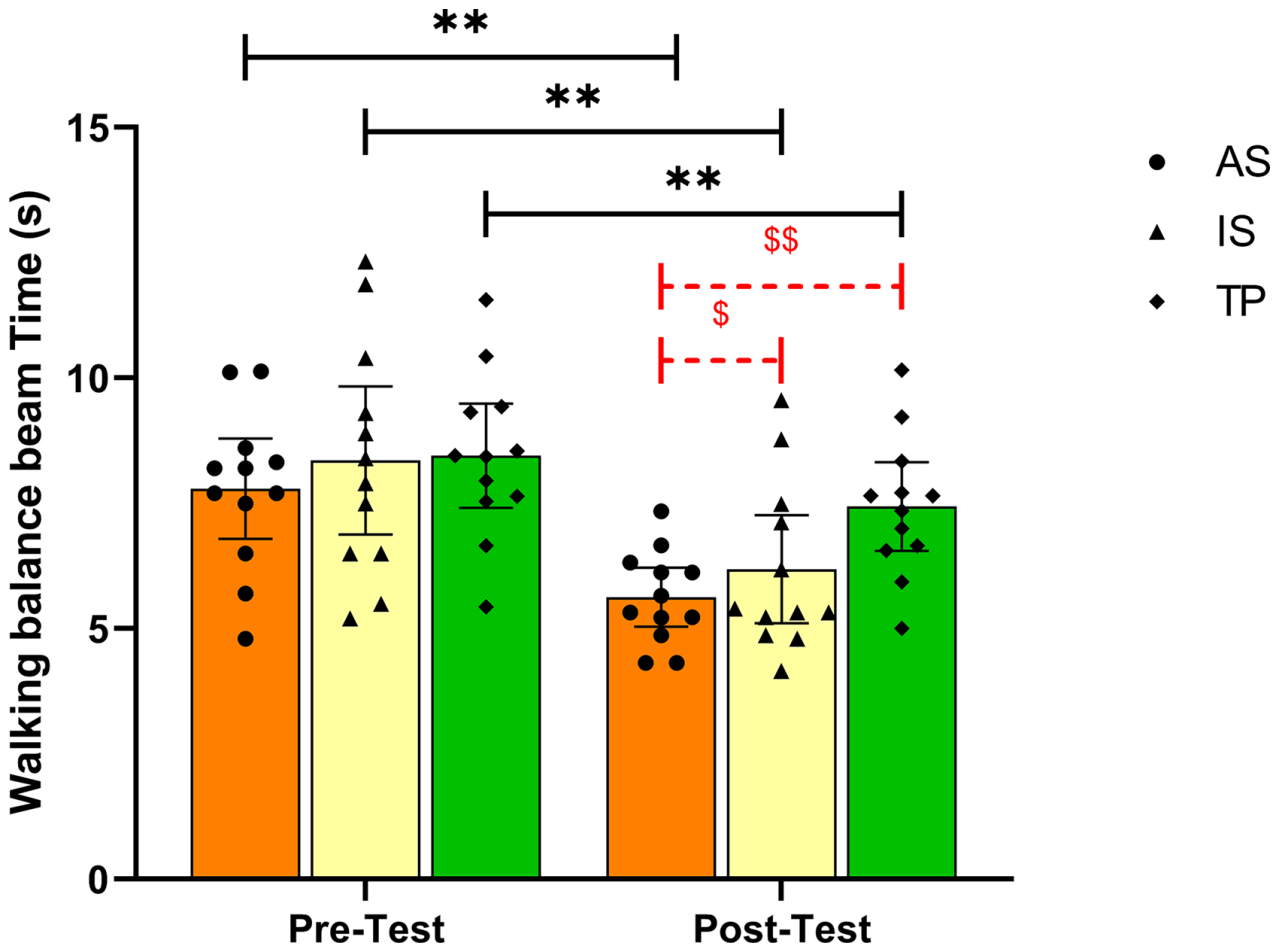
Effect of swimming on the walking balance beam time.*indicates a significant difference compared to the pre-test within the same group (*p < 0.05, **p < 0.01). S indicates a significant difference compared to TP group within the same time ($*p* < 0.05, $$*p* < 0.01). TP, traditional physical exercise group; AS, accompanied swimming group; IS, independent swimming group.

As shown in Figure 2, Bonferroni *post-hoc* comparisons showed time spent walking the balance beam was significantly lower in the AS group than in the TP group by 1.81 s (*p* < 0.01, [95% CI −3.22 to −0.40], ES = 1.53); time spent walking the balance beam was significantly lower in the IS group than in the TP group by 1.25 s (*p* < 0.05, [95% CI −2.66 to 0.16], ES = 0.81). As shown in Table 6, all groups showed significantly better fitness test levels following the physical exercise intervention (*p* < 0.01).

**Table 6:** Effect of swimming on the physical capacity.

Indicators	Time	TP (*n* = 12)	IS (*n* = 12)	AS (*n* = 12)
		M ± SD	M ± SD	M ± SD
Height (cm)	Pre	105.99 ± 4.59	106.92 ± 4.96	107.29 ± 3.80
	Post	107.84 ± 3.80^**^	108.83 ± 5.52^**^	108.86 ± 3.92^**^
Weight (kg)	Pre	18.29 ± 1.45	18.33 ± 1.78	18.39 ± 1.43
	Post	19.89 ± 1.38^**^	19.45 ± 1.51^**^	19.55 ± 1.67^**^
10-meter shuttle run time (s)	Pre	8.65 ± 1.09	9.27 ± 1.42	9.36 ± 1.09
	Post	7.90 ± 1.08^**^	8.20 ± 1.60^**^	8.64 ± 1.24^**^
Standing long jump (cm)	Pre	77.07 ± 6.86	75.33 ± 13.47	73.52 ± 13.07
	Post	82.25 ± 6.22^**^	85.75 ± 12.84^**^	83.33 ± 13.14^**^
Tennis ball throwing distance (m)	Pre	3.33 ± 0.60	3.19 ± 0.56	3.20 ± 0.60
	Post	3.87 ± 0.64^**^	4.15 ± 0.60^**^	4.28 ± 0.68^**^
Two-legged continuous jump time (s)	Pre	7.34 ± 1.72	8.46 ± 1.49	8.12 ± 2.09
	Post	6.67 ± 1.52^**^	6.50 ± 1.20^**^	6.50 ± 1.83^**^
Sit and reach (cm)	Pre	7.53 ± 1.33	7.02 ± 2.06	7.28 ± 1.84
	Post	8.44 ± 1.29^**^	8.86 ± 1.96^**^	9.47 ± 1.70^**^

^*^ indicates a significant difference compared to the pre-test within the same group (^*^p < 0.05, ^**^p < 0.01).TP, traditional physical exercise group; AS, accompanied swimming group; IS, independent swimming group.

### 3.2 Results of the effect of swimming exercise on IQ

All data in each group obeyed a normal distribution by the Shapiro–Wilk test (*p* > 0.05). The results of the spherical test showed that Machly W = 0.751, *p* = 0.02, which did not meet the spherical test. The results were calibrated in one-way ANOVA “Greenhouse–Geisser.” A one-way ANOVA test found homogeneity among the three groups in Pre-test (*p* > 0.05).

The results of the two-factor repeated measures ANOVA showed a significant time * group interaction effect (*F* = 26.253, *p* < 0.001, η^2^ = 0.455), a significant time main effect (*F* = 252.580, *p* < 0.001, η^2^ = 0.800) and a significant group main effect (*F* = 4.626, *p* = 0.013, η^2^ = 0.128). As shown in Table 7, Bonferroni *post-hoc* comparisons revealed that the TP group had lower IQ scores than the AS group at the mid-test (*p* < 0.05, [95% CI −12.45 to −0.96], ES = 0.89), and the TP group had significantly lower IQ scores than both the AS group (*p* < 0.01, [95% CI −14.12 to −2.74], ES = 1.15) and the IS tour group (*p* < 0.01, [95% CI −12.53 to −3.31], ES = 1.21) at the post-test. Pre-test IQ scores were significantly lower than mid-test and post-test (*p* < 0.01) across all three groups, and mid-test IQ scores were also significantly lower than post-test (*p* < 0.01).

**Table 7:** Effect of swimming on the intelligence quotient (points).

Group	Pre-test	Mid-test	Post-test
TP (n = 12)	104.08 ± 7.11	105.70 ± 7.58^**^	108.90 ± 6.68*^*##^
IS (n = 12)	106.17 ± 5.69	108.30 ± 5.49^**^	113.82 ± 6.36*^*##$$^
AS (n = 12)	104.83 ± 6.75	110.42 ± 7.68^**$^	114.33 ± 7.90^**##$$^

^*^indicates a significant difference compared to the pre-test within the same group (^*^p < 0.05, ^**^p < 0.01).^#^indicates a significant difference compared to the mid-test within the same group (^#^*p* < 0.05, ^##^*p* < 0.01).^$^indicates a significant difference compared to TP group within the same time (^$^*p* < 0.05, ^$$^*p* < 0.01).TP, traditional physical exercise group; AS, accompanied swimming group; IS, independent swimming group.

## 4 Discussion

Our main contribution in this study is to provide insights into the effects of 8 weeks of parent-accompanied swimming on the physical capacity and IQ scores of boys aged 3–4 years. This study showed that any form of exercise effectively improved the physical capacity of preschool boys. Swimming has a better effect on the intellectual level of preschool children than traditional land-based physical exercise.

Physical capacity is highly influenced by genetic factors and to some extent indicates the development of the preschool children’s bones, muscles, and internal organs. The results of this study demonstrated that physical exercise can promote growth in strength, flexibility, agility, coordination, and balance (24). We found that children’s tennis ball throw and standing long jump distance increased, which means their muscular strength and coordination improved to some extent after the 8-week exercise. Research has demonstrated that consistent, weekly physical exercise among children period can effectively increase their physical capacity levels and promote improvements in muscle strength and bone mass in the future (15). With approximately 800 times greater density than air, water can be used as a resistance medium to develop muscle strength in children without overloading their joints. For instance, a randomized controlled trial indicated that virtual treadmill training effectively enhanced school-age children’s gait, muscle strength, and gross motor function (26). To a certain extent, the 10-meter shuttle run time can reflect the oxygen transport ability of children’s blood at a younger age. Our findings indicate that physical exercise training efficiently enhances children’s running ability. Swimming has been shown to promote better cardiorespiratory fitness in children than other forms of exercise. For instance, Beggs et al. (27) found that swimming training had a meaningful effect on physical capacity, such as a mean increase in maximal oxygen uptake of 9.67 mL/kg/min. This is possibly due to increased hemodynamic gains caused by physical exercise. Lambrick et al. demonstrated that regular exercise increases oxygenated hemoglobin levels, which enhance executive function during exercise and, consequently, improve performance (28).

Our study found improvements in the time taken for two-legged continuous jumps and walking the balance beam, qualities that reflect balance and agility. Although direct evidence from studies in young children is limited, exercising may improve sensitivity and flexibility in adolescents (29). We also found that the time spent walking the balance beam was significantly lower in the swim training group than in the TP group. Similar result was obtained by Baccouch et al. in a randomized controlled trial of 36 preschool children aged 5–6 years, where swimming training was associated with better balance and motor skills than tennis training (30). Sigmundsson et al. (8) demonstrated that the benefits of learning to swim in preschool children facilitated the development of hand-eye coordination and stimulation of vestibular functions, which in turn led to an improvement in their balance. Aquatic training is now being utilized in the treatment of unwell children to enhance their neuromuscular functioning (31). The similar changes the children underwent during this study may have been linked to improved neuromuscular function. The enhanced muscle control of the children resulted in better balance. Swimming training involves children performing physical exercise in water that offer considerable resistance to motion. This type of resistance training enhanced the children’s explosive power and neuromuscular function (32). We also found it interesting that swimming with parental accompaniment can improve young children’s balance. Although it is challenging to identify comparable studies on preschoolers, a study conducted by Wilczyńska et al. yielded comparable outcomes in a cohort of child athletes. The results demonstrated that child athletes exhibited enhanced postural stability and balance when accompanied by coaches (33). Many factors can affect balancing capacity, such as taking medications, pain, fatigue, depression, anxiety, as well as auditory, visual and sensory stimuli and distractions. We speculated that this change could be related to psychological factors since parental accompaniment fosters a child’s well-being and, to some extent, stimulates an inclination toward learning the physical movements themselves (34). This phenomenon has been observed in children aged between five and seven in Finland, where family factors appear to significantly influence children’s ability to perceive movement (35). Numerous confounding factors must be considered to determine the direct impact on the physical capacity and motor performance metrics of young children. Further experimental research will be necessary to investigate this in the future.

Sufficient physical exercise during preschool guarantees that children’s brains are well-developed and helps their cognitive ability develop further (36). The C-WYCSI contains a standard assessment of a child’s verbal and operational IQ, quantifying the child’s cognitive ability level (25). This instrument has been utilized in various previous studies (37, 38). According to our findings, the IQ scores of children can be enhanced through any form of exercise, which also responded to an improvement in the subjects’ cognitive ability. Previous studies have indicated that physical exercise can influence brain function in the cognitive domain. For instance, Silva et al. (39) demonstrated enhanced cognitive flexibility in adolescents with ADHD before and after swimming training. A meta-analysis of children has identified a positive correlation between consistent physical exercise and cognitive ability development in children (40). Improvements in cognitive functioning play an important role in children’s ability to learn (41). Khawla et al. (42) concluded that 8 weeks of aerobic dance training improved executive functioning in children and that physical exercise is essential for children. It is widely believed that regular exercise can enhance the structure of the human brain and promote better brain development. Additionally, it can prevent unnecessary loss of brain cells, leading to improved cognitive performance. Researchers have observed a similar trend in experimental animals (43). Moderate physical exercise enhances the expression and concentration of brain-derived neurotrophic factors in the hippocampus (44, 45), thereby improving cognitive performance levels. The results of our experiments suggest that planned and regular swimming training better stimulate the development of children’s nervous, muscular, and other motor systems when compared to ordinary forms of training. Borioni and colleagues discovered that 10 times swimming sessions were adequate to enhance infants’ motor and cognitive functioning, implying that swimming is an effective strategy for improving cognitive flexibility in children (19). Swimming, as an aerobic activity, is an effective method of improving the cardiorespiratory endurance of children. It is hypothesized that this enhanced physical capacity may benefit intellectual development, leading to a further increase in IQ levels, which has been documented as a specific “mediating effect” by Ram-Tsur et al. (20). This is supported by the results of existing studies. Buck et al. (46) confirmed that higher aerobic capacity is usually associated with higher performance on the Stroop task, reflecting a link between motor and cognitive functioning. Verret et al. also found that children with ADHD all scored significantly lower in motor skills, indirectly confirming this ‘mediating effect’ (47). Definitely, it is also possible that the subjects’ familiarity with the testing process, as a result of the multiple testing sessions, subsequently enhanced the IQ test scores. The study found that swimming exercises with parental accompaniment for a four-week duration proved more effective in enhancing children’s cognitive abilities compared to independent swimming. For children to develop and comprehend their surroundings, they require parental support, and to facilitate cognitive growth, they necessitate continuous interaction with the environment, using parental figures as the point of departure. Research has indicated that physical interaction between children and their guardians in the water is efficacious in advancing brain and nervous system growth, thus boosting cognitive function (48). Furthermore, the behaviors and perceptions children acquire are often influenced by their unique experiences at individual, parental, and familial levels. In this regard, the swim-exercise model, which involves parental supervision, can effectively enhance young children’s motor learning and control (49).

Our study yielded some results on the effects of swimming exercise on children’s physical capacity and intelligence, but there are still some limitations. First, the study included only boys, neglecting gender differences. Previous evidence suggests that dichotomous results may differ between boys and girls due to differences in the gonadal axis (50), which needs to be further improved in the future; secondly, we neglected factors such as family background and parents’ literacy level, which need to be further considered in the future; lastly, this study only collected indicators of physical training and IQ tests, which does not explain the changes of exercise stimulation on the physiological level for children. More physiological indicators such as maximum oxygen uptake, blood routine, EEG signals, etc. should be considered to collect in future studies. This will help us provide corresponding answers from the perspective of physiological mechanisms.

## 5 Conclusion

In this study, a twice-weekly physical exercise program over 8 weeks was found to improve physical capacity and IQ scores in preschool children. Additionally, swimming was more beneficial for balance and IQ than traditional physical exercise. Finally, swimming with parents improved IQ more than swimming independently over 4 weeks. These findings suggest that the inclusion of swimming exercises in physical exercise regimes for younger children is crucial. Designing appropriate exercise programs for children can significantly enhance their physical capacity and cognitive abilities.

## Data availability statement

The original contributions presented in the study are included in the article/supplementary material, further inquiries can be directed to the corresponding authors.

## Ethics statement

The study adhered to the Declaration of Helsinki, and the Ethics Committee of Exercise Science at Beijing Sport University approved the study protocol (Grant no. 202008211). The studies were conducted in accordance with the local legislation and institutional requirements. Written informed consent for participation in this study was provided by the participants’ legal guardians/next of kin.

## Author contributions

YY: Conceptualization, Data curation, Formal analysis, Investigation, Software, Validation, Visualization, Writing – original draft, Writing – review & editing. LX: Conceptualization, Data curation, Formal analysis, Investigation, Software, Validation, Visualization, Writing – original draft, Writing – review & editing. HY: Conceptualization, Methodology, Project administration, Supervision, Validation, Writing – review & editing. YL: Conceptualization, Formal analysis, Methodology, Project administration, Resources, Supervision, Writing – review & editing.
